# Preparation and antibacterial and antioxidant ability of β‐cyclodextrin complexes of vaporized *Illicium verum* essential oil

**DOI:** 10.1002/fsn3.2997

**Published:** 2022-07-26

**Authors:** Kegang Wu, Tong Zhang, Xianghua Chai, Dong He, Xuejuan Duan, Bingying Yu, Yongqi Chen, Yuqiang Huang

**Affiliations:** ^1^ School of Chemical Engineering and Light Industry Guangdong University of Technology Guangzhou China

**Keywords:** antibacterial properties, antioxidant properties, encapsulation, *Illicium verum* essential oil

## Abstract

Compared with traditional liquid–liquid embedding method and solid–liquid embedding method of inclusion complexes of β‐cyclodextrin (β‐CD) inclusion of essential oil to form stable properties, the gas–liquid embedding method was applied to encapsulate vaporized *illicium verum* essential oil (IvEO), with β‐CD as wall materials so that core and wall materials molecules are in active state during complexing process. At optimal conditions with a mass ratio of 1:10, temperature of 80°C, time of 1 h, the β‐CD‐IvEO inclusion complexes (β‐CD‐IvEO‐ICs) had an encapsulation efficiency (EE) of 84.55 ± 2.31%. Fourier transform infrared spectroscopy (FTIR) revealed the encapsulation of IvEO into inclusion complexes, differential scanning calorimetry (DSC) and thermogravimetric analysis (TGA) demonstrated the enhanced thermal stability of IvEO after embedding. Furthermore, the reducing power and 2‐phenyl‐4,4,5,5‐tetramethylimidazoline‐1‐oxyl‐3‐oxide (PTIO)‐scavenging capacity displayed certain capacity of antioxidation in a short time but stronger antioxidative activities as reaction time was extended. The diameter of growth zone (DGZ) indicated stronger antibacterial activity of β‐CD‐IvEO‐ICs against *Escherichia coli*, *Bacillus subtilis*, *Staphylococcus epidermidis*, and *Staphylococcus aureus*. Moreover, the β‐CD‐IvEO‐ICs could induce the bacteria producing more reactive oxygen species (ROS) than IvEO, resulting in bacterial death.

## Graphical abstract

Compared with traditional liquid‐liquid enbedding method and solid‐liquid enbedding method of inclusion complexes of #x03B2;‐cyclodextrin (#x03B2;‐CD) inclusion of essential oil to form stable properties, gas‐liquid embedding method was applied to encapsulate vaporized illicium verum essential oil with #x03B2;‐CD (#x03B2;‐cyclodextrin) as wall materials so that core and wall materials molecules are in active state during complexing process. #x03B2;‐CD‐IvEO‐ICs showed stronger antibacterial ability against Escherichia coli, Bacillus subtilis, Staphylococcus#x00A0;epidermidis, Staphylococcus aureus.

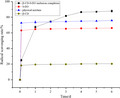

**Practical Application**

β‐CD‐*Illicium verum* essential oil inclusion complexes (β‐CD‐IvEO‐ICs) can prevent IvEO from being oxidized and increase its antibacterial activity so that β‐CD‐IvEO‐ICs could be employed as a potential natural preservative in food industry.

## 1 INTRODUCTION

*Illicium verum*, as a traditional herbal medicine, belongs to the Magnoliopsida family, which possesses effective antimicrobial, antioxidant, insecticidal (Ibrahim et al., 2019; Park et al., 2016), analgesic and sedative activities (Wang, Hu, et al., 2011). It is distributed widely in China, especially in the provinces of Guangxi, Fujian, Yunnan, Taiwan, Guangdong, and Guizhou (Cai et al., 2013). *I. verum* has long been used in traditional Chinese medicine and food industry. For instance, *I. verum* is the industrial source of shikimic acid, a primary ingredient to make antiviral drug Tamiflu, which is regarded as a remedy for the bird flu H5N1 strain of virus (Wang, Hu, et al., 2011). Additionally, it is commonly used as spice for its volatile oil in food industry, and it is often used as spice whose smell comes from its volatile oil. However, IvEO, which has poor water solubility, high volatility, unstable physicochemical properties, and unpleasant smell, shows a relatively low availability in the application of food industry.

Considering the limitation of IvEO, there are increasing researches on preparing inclusion complexes to improve its physical and chemical properties. Zhang et al. reported that selective encapsulation of star anise essential oil (SAEO) by hydroxypropyl‐β‐cyclodextrin could reduce the irritating smell of SAEO, and improve the inhibition effect of SAEO on *Rhizopus stolonifer*, *Saccharomyces cerevisiae*, and *E. coli* and its antibacterial stability (Zhang et al., 2018). IvEO could also be encapsulated by chitosan to enhance antifungal and antiaflatoxigenic potency (Dwivedy et al., 2018). The trans‐anethole/β‐cyclodextrin inclusion complexes could be evenly dispersed in the gelatin‐based edible films with appropriate addition, which improved the tensile strength and surface hydrophobicity and reduced the moisture content of the edible films (Ye et al., 2017). Therefore, suitable wall materials and effective methods could improve various properties of IvEO. β‐CD, as a wall material, has the advantages of masking undesired smell or taste, preventing essential oil from being oxidized (Hill et al., 2013; Kavetsou et al., 2021), and increasing the water solubility (Celebioglu et al., 2018) as well as being nontoxic and of low cost (Beirão da Costa et al., 2012).

The method of increasing diffusion forces between gaseous cinnamon essential oil and β‐CD solution so that core and wall materials molecules are in active state during complexing process has been studied (Zhang, 2017). Moreover, antibacterial and antioxidant properties of essential oils (EOs) before and after encapsulation have been extensively studied (Raksa et al., 2017). Nonetheless, the characterization of inclusion complexes that were prepared by the gas–liquid embedding method, antioxidant activity during sustained release process of inclusion complexes and its antibacterial mechanism have been rarely studied. Thus, this study aimed to prepare and characterize the β‐CD‐*Illicium verum* EO inclusion complexes (β‐CD‐IvEO‐ICs) and compare the different antioxidant activities between IvEO and β‐CD‐IvEO‐ICs through the ferric reducing antioxidant power (FRAP) assay and 2‐phenyl‐4,4,5,5‐tetramethylimidazoline‐1‐oxyl‐3‐oxide (PTIO)‐scavenging assay. Additionally, the long‐term antibacterial activity of IvEO and β‐CD‐IvEO‐ICs against *E. coli*, *B. subtilis*, *S. epidermidis*, and *S. aureus* was evaluated and compared.

## 2 MATERIALS AND METHODS

### 2.1 Materials

*Escherichia coli* (ATCC8739), *S. aureus* (ATCC65389), *B. subtilis* (ATCC6633), and *S. epidermidis* (ATCC12228) were purchased from the Guangdong Institute of Microbiology.

*Illicium verum* essential oil (IvEO) was obtained from Xiangsi Xinqing Health Technology Co., Ltd. Nutrient agar was supplied by Guangdong Huangkai Microbial Sci. &Tech. Co., Ltd. Total antioxidant capacity assay kit was supplied by Suzhou Grace Biotechnology Co., Ltd. Reactive oxygen species (ROS) assay kit was supplied by Applygen Technologies Inc., Beijing, China. PTIO• (CAS 18390‐00‐6) was supplied by Biohonor Technology Co., Ltd. All other agents used for experiment were of analytical grade. Deionized water was used to perform all experiments.

### 2.2 Preparation of β‐CD‐IvEO‐ICs

**FIGURE 1:**
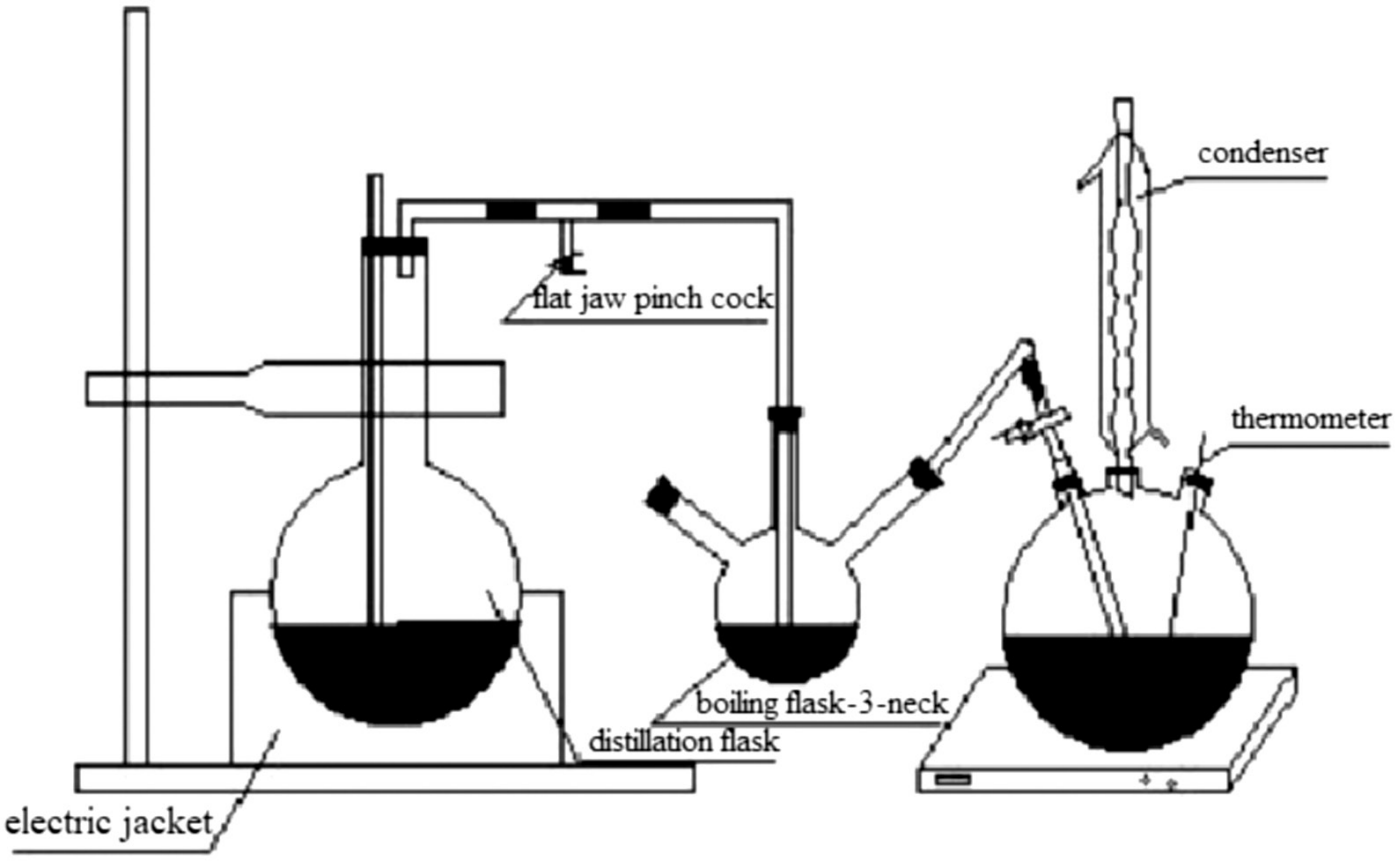
Gas–liquid embedding device.

The β‐CD‐IvEO‐ICs were prepared as previously described with slight modifications (Zhang, 2017). The β‐CD solution was prepared using hot distilled water with water:β‐CD ratio of 9:1. The extraction device is shown in Figure 1. High temperature steam, evaporated from distillation flask, was led to a 3‐neck boiling flask and the IvEO heated. After IvEO (core material) completely evaporated, it led into a β‐CD solution, and kept sealed – heating at a certain temperature with stirring for a certain time. The mixture was then kept stirring at 300 r min^−1^ and 25°C for 5 h and the complexes were dried at 40°C for 4 h after vacuum filtration. The β‐CD‐water‐ICs were prepared in the same way with water as the core material.

### 2.3 The effects of the preparation conditions on the EE

The inclusion complexes’ preparation conditions were controlled to evaluate the influences of the ratio of oil to wall materials, temperature, and time on the encapsulation efficiency (EE) through a single‐factor experiment (Cui et al., 2021).

#### 2.3.1 Ratio of oil to wall materials

The β‐CD‐IvEO‐ICs preparation conditions were controlled to evaluate the influences of the oil–wall ratio (1:4, 1:6, 1:8, 1:10, 1:12, 1:14) on the EE at 80°C and 300 r min^−1^ for 1 h.

#### 2.3.2 Temperature

The EE of β‐CD‐IvEO‐ICs was evaluated and the β‐CD‐IvEO‐ICs were prepared by the gas–liquid embedding method with the oil–wall ratio of 1:10 at different temperatures (50, 60, 70, 80, 90, and 100°C) and 300 r min^−1^ for 1 h.

#### 2.3.3 Time

The β‐CD‐IvEO‐ICs preparation conditions were controlled to evaluate the time condition (0.5, 1, 1.5, 2, 2.5, and 3 h) on the EE with the oil–wall ratio of 1:10 at 300 r min^−1^ for 1 h.

### 2.4 Orthogonal design

The ratio of oil to wall materials, time, and temperature conditions were selected as three influential factors (marked as A, B, and C). Influential factors were investigated at three levels. EE was used as the evaluation index to confirm the optimum process parameter.

### 2.5 Particles characterization

#### 2.5.1 Scanning electron microscopy (SEM)

After adhering to the sample stage and sprayed gold, the β‐CD‐water‐ICs and β‐CD‐IvEO‐ICs were analyzed using a scanning electron microscope (SEM) (Hitachi Regulus 8100, Hitachi, Ltd.) at various magnifications under high vacuum and 3.0 kV voltage (Natrajan et al., 2015).

#### 2.5.2 Fourier transform infrared spectroscopy

The Fourier transform infrared spectroscopic (FT‐IR) assay was conducted as described earlier with some modifications (Yang et al., 2021). The β‐CD, IvEO, β‐CD, and IvEO physical mixture and β‐CD‐IvEO‐ICs were mixed separately with potassium bromide (KBr) and abraded and pressed for slice formation. The spectra were obtained in the infrared (IR) region of 4000–400 cm^−1^ through Fourier transform infrared spectroscopy (FTIR) (Nicolet 6700 FTIR, Thermo Fisher Scientific), with a scanning number of 32 and resolution of 1 cm^−1^.

#### 2.5.3 EE determination

The IvEO content was determined using a spectrophotometer according to the method described previously with some modifications (Ghazy et al., 2021; Zhang, 2017). Different concentrations of IvEO standard solutions (1, 2, 3, 4, 5, and 6 μl L^−1^) were prepared using absolute ethanol, and absorbance of the solutions was measured at 259 nm with absolute ethanol as the blank control. The IvEO concentration and absorbance were taken as the abscissa and ordinate, respectively. The obtained standard calibration curve equation was: *Y* = 0.1259*x* − 0.0014, *R* ^2^ = 0.9998.

The inclusion complexes particles (0.02 g) were added into a 10 ml volumetric flask and constant volume with ethanol. Surface oil in the inclusion complexes was washed by 10 ml ethanol. The total oil of β‐CD‐IvEO‐ICs was dissolved using 1 ml hot water and 200 μl absolute ethanol with stirring at 300 r min^−1^ for 2 min. Absorbance values of all the solutions were determined using an ultraviolet–visible (UV–Vis) spectrophotometer (TU‐1950, Beijing Purkinje General Instrument Co., Ltd.) at 259 nm. The amount of IvEO was estimated using the standard calibration curve. The EE was calculated as following Equation (1):

(1) $$\mathrm{EE}，\%=\left(1-W_{1}\right)/W_{2}\times 100$$

where *W*_1_ is the mass (g) of oil on the β‐CD‐IvEO‐ICs surface and *W*_2_ is the total mass (g) of oil into β‐CD‐IvEO‐ICs.

#### 2.5.4 Differential scanning calorimetry (DSC) and thermogravimetric analysis (TGA)

Thermal stability of the β‐CD, IvEO, β‐CD, and IvEO physical mixture and β‐CD‐IvEO‐ICs was determined using a STA449F5 thermal analyzer (Netzsch). All materials (3 mg) were added into the sample room and subjected to heating from 30 to 1000°C at 10°C min^−1^ heating rate under nitrogen atmosphere (Liu et al., 2015; Yang et al., 2021).

### 2.6 Antioxidative assay

#### 2.6.1 Determination of ferric reducing antioxidant power (FRAP assay)

In brief, freshly prepared FRAP reagent was warmed to 37°C. After 10 μl IvEO/β‐CD equivalents of the samples, 90 μl 80% ethanol and 100 μl ultrapure water were added to 850 μl FRAP reagent, the mixture was incubated at 25°C for different times (10 min and 1 h). Absorbance values of all the solutions were determined using a UV–Vis spectrophotometer (TU‐1950, Beijing Purkinje General InstrumentCo., Ltd.) at 590 nm. The reducing power of samples was estimated using the standard calibration curve, and the results are presented as Trolox equivalent antioxidant capacity (μmol Trolox 100 g^−1^) (Cuong et al., 2020).

#### 2.6.2 2‐Phenyl‐4,4,5,5‐tetramethylimidazoline‐1‐oxyl‐3‐oxide (PTIO·)‐scavenging activity measurements of IvEO and IvEO‐NPs

The methodology for determining the radical‐scavenging ability referred to Li et al. with slight modifications (Li et al., 2021). In brief, 10 μl IvEO/β‐CD equivalents of IvEO, β‐CD‐IvEO‐ICs, β‐CD, and physical mixture were prepared with 750 μl deionized water and 1.2 mg ml^−1^ PTIO solution was prepared with deionized water. One thousand 1000 μl of the oil solutions was mixed with 90 μl PTIO soution with essential oil free as blank control. Absorbance of samples and controls was measured at 557 nm after mixing and incubation in dark at 25°C for different times (2 h, 1 day, 2 days, 3 days, 4 days, 5 days, and 6 days) with TU‐1950 UV–Vis spectrophotometer. The percent inhibition of the PTIO radical by the samples was calculated according to Equation (2):

(2) $$\%\text{Inhibition}=\left(1-\frac{A_{\text{reaction mixture}}-A_{\text{sample}}}{A_{\text{blank}}}\right)\times 100$$

where *A*_blank_ is absorbance without sample, *A*_reaction mixture_ is absorbance with sample, and *A*_sample_ is absorbance with sample.

### 2.7 Antimicrobial assay

#### 2.7.1 Microorganisms

Strains were streaked on Nutrient agar plates and placed in a 37°C incubator for 24 h. A single colony was picked from the plate and bacterial suspension with absorbance of 3.0 at 600 nm was prepared with TU‐1950 UV–Vis spectrophotometer.

#### 2.7.2 Growth zone measurement

The zones of growth for bacteria under IvEOs treatment were determined using the Oxford cup plate assay. The methodology was used with some modification in the method described earlier (Yang et al., 2021), which is adding bacterial suspension into Oxford cups to make it grow and divide on the IvEOs plates. A study showed that agar is generally formed as a polyelectrolyte gel and the resulting 3D polymeric network has porous structure, which allows the liquid to diffuse and predicted that essential oil in a Petri dish might be relevant to its diffusion coefficient into agar medium (Mutlu‐Ingok et al., 2020). Because of the low diffusion rate of EO, adding EO into Oxford cups may result in a phenomenon that bacteria have already grown on the surface before EO diffuses into the surface of agar plates, which causes a poor antibacterial effect. Therefore, Oxford cups were always taken out after the medium solidified, and then the antibacterial substance was loaded into the holes so that it can come into contact with the bacteria directly according to a study (Yang et al., 2018). On the contrary, bacteria could grow normally in the agar by adding bacterial suspension into cups and antibacterial substances have already diffused before exposing to bacteria, which means it offers no length of diffusion time issues for antibacterial substances. Subsequently, two treatment groups were designed to evaluate the antibacterial activity of IvEO and β‐CD‐IvEO‐ICs. The IvEO concentration of the β‐CD‐IvEO‐ICs groups was adjusted to that of the IvEO groups.

IvEO and β‐CD‐IvEO‐ICs were added into Nutrient agar (cool to 40°C) respectively. First, 10 ml of molten Nutrient agar was added into sterilized plates until solidification. Then, another 10 ml of molten Nutrient agar was poured on the plates. Afterwards, two Oxford cups were placed on each Nutrient agar plate. After cooling and solidification of Nutrient agar, each bacterial suspension was added into one of the cups and sterile water was added into another cup as a negative control. The culture dishes were incubated at 37°C for 1, 2, 3, 4, 5, 6, 7, 8, 9, and 10 days to observe differences in strain growth in IvEO and β‐CD‐IvEO‐ICs plates. The diameter of each inhibition zone was measured using cross method. The antibacterial activity of IvEO and β‐CD‐IvEO‐ICs was estimated by measuring the diameter of growth zone (DGZ).

#### 2.7.3 Detection of ROS

In brief, 20 ml of molten Nutrient agar (cool to 40°C) was mixed with certain amount IvEO and β‐CD‐IvEO‐ICs and poured on the plates with sterile water as blank control. Then, the bacterial suspension (50 μl) with absorbance of 3.0 at 600 nm was inoculated onto Nutrient agar plates in a 37°C incubator for 24 h. Afterwards, the treated bacteria were collected by washing the plate using 5 ml phosphate‐buffered saline (PBS). After the suspension adjusted to the absorbance of 0.200 ± 0.005 at 600 nm then triple diluted, 3 μl of 10 mmol L^−1^ dichlorodihydrofluorescein diacetate (DCFH‐DA) was added into the mixture in dark at 37°C for 30 min and the mixture was shaken every 5 min.

The dichlorofluorescein (DCF) fluorescence of the mixture mentioned above was measured using a fluorescence spectroscope (Fluorolog‐3, HORIBA Instruments Incorporated) with excitation wavelength of 480 nm, emission wavelength of 525 nm, and slit width of 1 nm.

### 2.8 Statistical analysis

All experiments were performed at least in triplicate and data are expressed in means ± standard deviations (*n* > 3). Statistical analyses were performed using Origin 2018.

## 3 RESULTS AND DISCUSSION

### 3.1 Effects of preparation conditions on the EE

#### 3.1.1 Ratio of oil to wall materials

**FIGURE 2:**
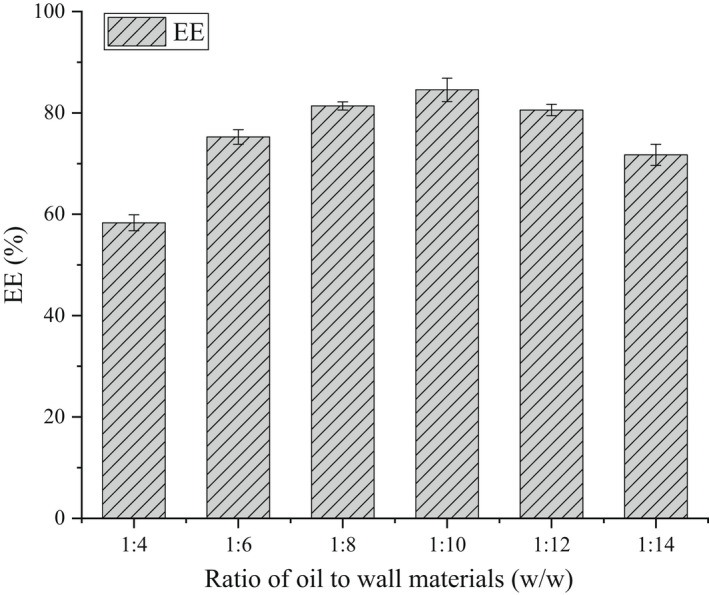
Effect of the ratio of oil to wall materials on the encapsulation efficiency (EE).

As shown in Figure 2, the yield of β‐CD‐IvEO‐ICs increased significantly, reaching the highest value at the oil:wall material mass ratio of 1:10 (w/w), followed by a decrease. At high concentration of oil material, the free oil adhered to the surface of β‐CD‐IvEO‐ICs, which causes their aggregation to lower the EE. Increasing the ratio of wall material, which means more IvEOs can be fully immersed in the hydrophobic cavity of β‐CD, could gradually increase the EE which is consistent with previous studies. However, the saturation of β‐CD by further embedding of IvEO would decrease the EE of inclusion complexes. This was probably due to a dynamic equilibrium during complexing process between β‐CD and IvEO in aqueous solutions. The equation of dissociation and inclusion was shown as follows (Equation [3]):

(3) β−CD−IvEO⇌β−CD+IvEO

Namely, such an encapsulation method resulted in the encapsulation efficiency that cannot reach 100%. The result was also observed in a study, which showed that some content of a core material can be found on the outer surface or some areas that are close to the surface of an encapsulate at a higher load (Jafari et al., 2008). According to a report, the hydrophobic cavity that wall material provided during the complexing process is not completely occupied, which means the relationship of nonstoichiometric host molecule and guest molecule is available (Sun, 2012). It can be considered that the complexing process has achieved equilibrium at an oil:wall material mass ratio of 1:10 (w/w), which means there is not enough essential oil for further inclusion with the mass of wall material increases. After that, the mass of free oil can be considered as an invariable constant. According to Equation 3, on the basis of invariable mass of free oil, decreasing the mass of whole essential oil can decrease the EE, which causes the EE of complexing process to hardly reach 100% in aqueous solutions.

#### 3.1.2 Temperature condition

**FIGURE 3:**
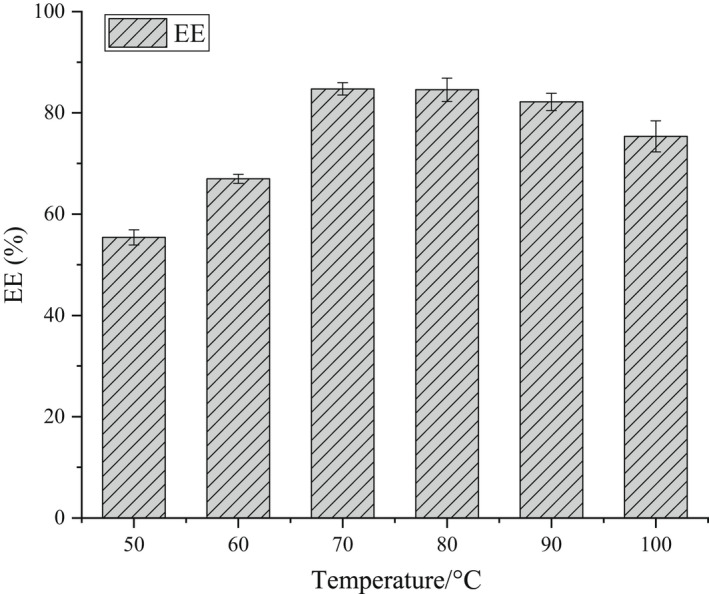
Effect of the temperature condition on the encapsulation efficiency (EE).

Figure 3 shows the influence of temperature on the EE of β‐CD‐IvEO‐ICs. The EE of β‐CD‐IvEO‐ICs increased with increase of temperature from 50 to 70°C. This was probably due to the low reaction rate under low temperature during the complexing process. Meanwhile, it is hard for IvEO which has low solubility to have adequate contact with β‐CD molecule at low temperature. On the contrary, an increase in temperature can accelerate the dissolution of β‐CD and increase the impact rate of IvEO molecule for the β‐CD molecule thus promoting self‐assembly.

When the temperature value ranged from 80 to 100°C, the EE of β‐CD‐IvEO decreased gradually probably due to anethole, as the main ingredient of IvEO, which could gradually react with oxygen at 55 ~ 95°C and increase its oxidation reactivity which lead to the reduction of oil material (Zhang, 2018).

#### 3.1.3 Time condition

**FIGURE 4:**
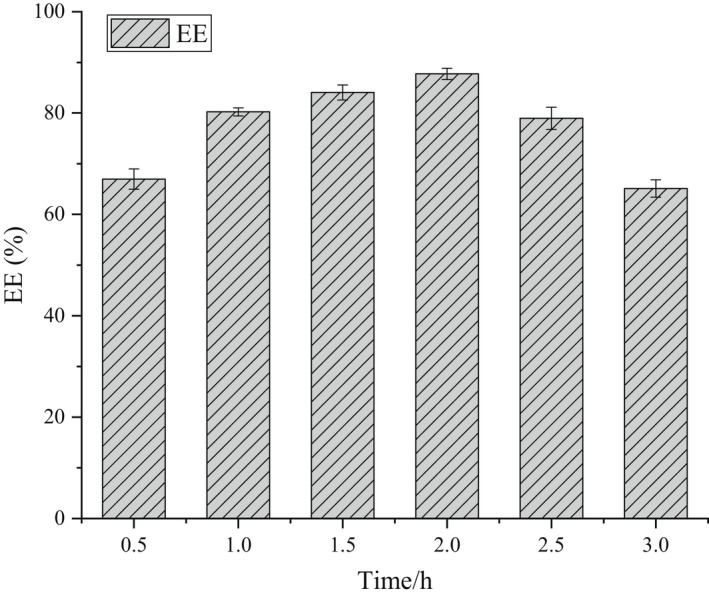
Effect of the time condition on the encapsulation efficiency (EE).

Figure 4 represents the effect of different reaction times on the EE of β‐CD‐IvEO‐ICs. The EE values initially increased gradually and then dropped significantly. This was due to the fact that the shortened reaction time could cause an inadequate contact between IvEO molecules and β‐CD molecules. Moreover, the oxidation process of anethole can change from oxygen absorption stage to rapid oxidation stage with increased reaction time, which caused the decreased mass of oil material (Zhang, 2018). It can be stated that excessive reaction time may lower the EE.

### 3.2 Analysis of the orthogonal design

As shown in Table 1, the *R*‐value decreased in the order: *R*_*B*_ > *R*_*A*_ > *R*_*C*_. Accordingly, the influence of each factor on EE in decreasing order was as follows: time value > the ratio of oil to wall materials > temperature value. Obviously, the time value had great influences on the EE of β‐CD‐IvEO‐ICs. When the mass ratio of oil to wall materials, time, and temperature conditions were 1:10 (w/w), 1 h, 80°C, respectively, the EE reached 84.55 ± 2.31%. The EE of verification testing was 85.59%，which showed that the process is reasonable and feasible.

**TABLE 1:** The design and results of the orthogonal formulation

Numbers/factors	A	B	C	D	EE%
1	1:8	1 h	70°C	1	78.17 ± 1.11
2	1:8	1.5 h	80°C	2	84.04 ± 1.47
3	1:8	2 h	90°C	3	61.97 ± 1.38
4	1:10	1 h	80°C	3	84.55 ± 2.31
5	1:10	1.5 h	90°C	1	80.42 ± 1.06
6	1:10	2 h	70°C	2	79.50 ± 0.86
7	1:12	1 h	90°C	2	80.27 ± 0.87
8	1:12	1.5 h	70°C	3	75.54 ± 2.04
9	1:12	2 h	80°C	1	62.87 ± 1.29
*k*1	74.727	80.997	77.737	73.820	
*k*2	81.490	80.000	77.153	81.270	
*k*3	72.893	68.113	74.220	74.020	
*R*	8.597	12.883	3.517	7.450	

*Note*: A means the ratio of oil to wall materials, B means the time values, C means the temperature values, and D means blank column. *ki*1 is the mean values for level 1 of each factor; *ki*2 is the mean values for level 2 of each factor; *ki*3 is the mean values for level 3 of each factor. *R* is the difference between the maximun and minimum of the encapsulation efficiency (EE) for every level. *R* = max (*kij*)–min (*kij*).

### 3.3 β‐CD‐IvEO inclusion complexes characterization

#### 3.3.1 SEM

**FIGURE 5:**
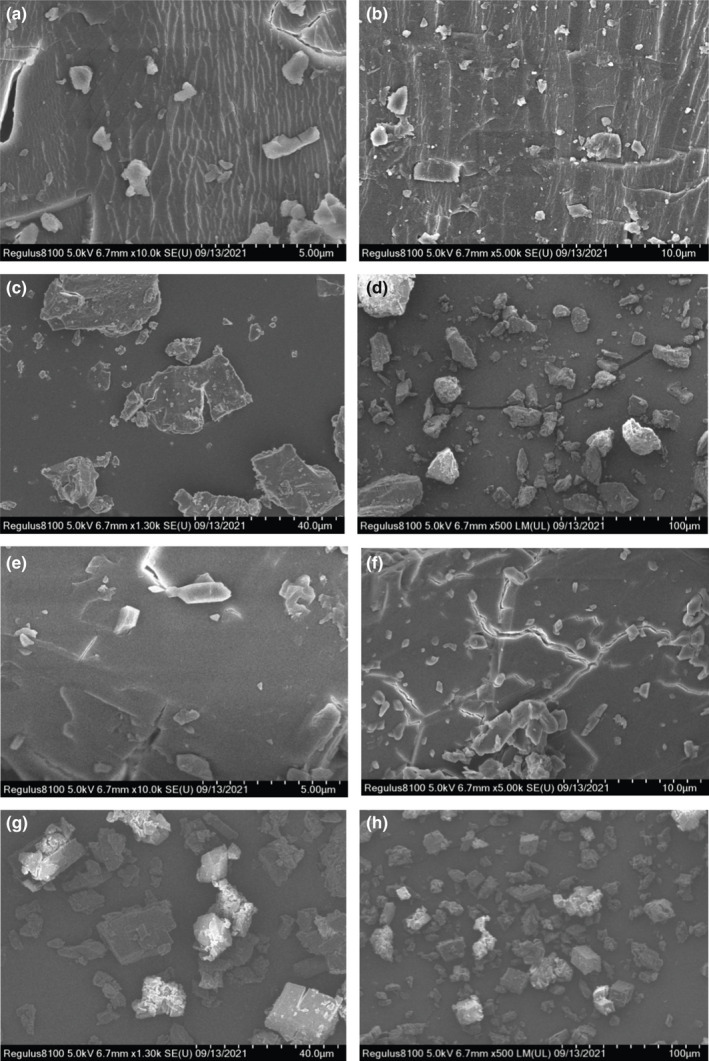
Scanning electron microscopy (SEM) micrographs of inclusion complexes (ICs); (a) β‐cyclodextrin (β‐CD)‐water inclusion complexes × 10,000；(b) β‐CD‐water inclusion complexes × 5000; (c) β‐CD‐water inclusion complexes × 1250; (d) β‐CD‐water inclusion complexes × 500; (e) β‐CD‐IvEO inclusion complexes × 10,000; (f) β‐CD‐IvEO inclusion complexes × 5000; (g) β‐CD‐IvEO inclusion complexes × 1250; (h) β‐CD‐IvEO inclusion complexes × 500.

It can be noted in Figure 5(a,b,e, and f) that β‐CD‐water‐ICs were regular, translucent block, and cracked. β‐CD‐IvEO‐ICs were amorphous fine particle and crack‐free. As shown in Figure 5(c,d,g, and h), β‐CD‐water‐ICs were compact and big crystal. However, β‐CD‐IvEO‐ICs was small aggregate with lots of particulate by loose conglomeration, which was obviously different from β‐CD‐water‐ICs. According to a report, β‐CD‐water‐ICs consisted solely of β‐CD molecule, in which intramolecular or intermolecular hydrogen bonds between the C2 and C3 hydroxyl form hydrogen‐bond‐type crystal lattice (Prabhu et al., 2018). After β‐CD forms inclusion with IvEO molecule, the IvEO molecule can interfere with the formation of hydrogen bonds, and change the molecular arrangement or accumulation to affect changes in crystal morphology. The results are in line with those of a study (Hua.Chai et al., 2011).

#### 3.3.2 FTIR features measurement

**FIGURE 6:**
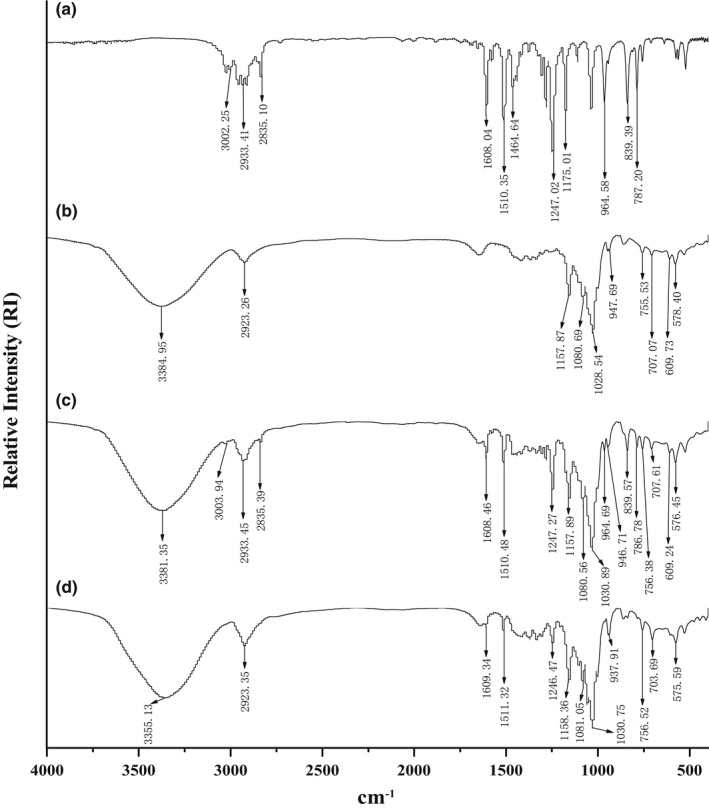
The Fourier transform infrared spectroscopy (FTIR) of *illicium verum* essential oil (IvEO) (a), β‐cyclodextrin (β‐CD) (b), physical mixture (c), and β‐CD‐IvEO‐ICs (inclusion complexes) (d).

The FTIR was applied to evaluate the EE based on the structure and the interactions. Differences in the structures of IvEO (a), β‐CD (b), physical mixture (c), andβ‐CD‐IvEO‐ICs (d) are illustrated in Figure 6. Both the characteristic peaks of the IvEO Figure 6(a) and β‐CD Figure 6(b) covered the field of 400–4000 cm^−1^. The vibration in (a) occurring arround 3002.25 and 964.58 cm^−1^ derived from = C‐H, C=C of IvEO, respectively, 1608.04, 1510.35, and 1464.64 cm^−1^ from the aromatic group of IvEO, 1247.02 cm^−1^ from the aromatic ether of IvEO, 839.39 cm^−1^ from 1,4‐substituted benzene, and 2933.41 cm^−1^, 2835 cm^−1^ from −CH_3_ and −CH_2_ groups of IvEO, respectively. For β‐CD, the absorption peak at 3384.95 cm^−1^ was attributed to the vibration absorption peak of −OH, and the absorption peak at 2923.26, 1157.87, and 1028.54 cm^−1^ corresponded to the vibrations of the C‐H, C‐O, and C‐O‐C groups of IvEO. Combined with (a) and (b), (c) had distinct absorption peaks that derived from IvEO, which appeared at 3003.93 cm^−1^ (=C‐H), 2933.45 cm^−1^, 2835.39 cm^−1^ (C‐H), 1608.46 cm^−1^, 1510.48 cm^−1^ (aromatic group), 1247.47 cm^−1^ (aromatic ether), 964.69 cm^−1^ (C=C), and 839.57 cm^−1^ (1,4‐substituted benzene). Regarding β‐CD‐IvEO‐ICs, the observed absorption peak of O‐H of β‐CD had moved to a high wave number direction (3355.13 cm^−1^), which could be related to the formation of intermolecular hydrogen bonds. Additionally, compared with (c), the typical intense peaks of 1246.47, 756.38, and 575.59 cm^−1^ were weakened and 3003.94, 2835.39, 964.69, 839.57, and 786.78 cm^−1^ could not be observed in (d). The disappearance of typical peak indicated that the aromatic ring of IvEO was included in the β‐CD cavity. This restricted molecular vibration suggested the successful formation of the β‐CD‐IvEO‐ICs as reported by Haiyee et al. (2009). In addition, inclusion complexes, β‐CD and physical mixture had great difference between 1200 and 1600 cm^−1^ which display the variation of C‐H absorption (Wang et al., 2011b). This was probably due to the fact that IvEO molecules combined with the hydrophobic bond of β‐CD molecules (Haiyee et al., 2009) under the impetus of van der Waals force and hydrophobic interaction (Yang et al., 2009), which promoted the formation of β‐CD‐IvEO‐ICs.

#### 3.3.3 DSC analysis

**FIGURE 7:**
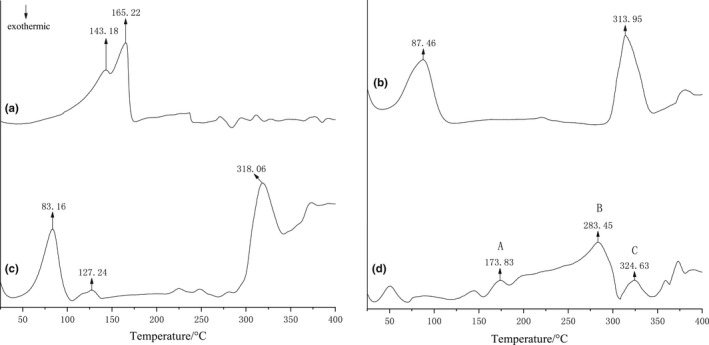
Differential thermal analysis curves of *illicium verum* essential oil (IvEO), β‐cyclodextrin (β‐CD), physical mixture, and β‐CD‐IvEO‐ICs (inclusion complexes).

When guest molecules were embedded into β‐CD cavities, their melting, boiling, or sublimating points generally shifted to different temperatures or disappeared (Wang et al., 2011a). Thus, DSC can be used for the recognition of inclusion complexes. As shown in Figure 7, two endothermic peaks observed at 143.18 and 165.22°C for IvEO were related to its endothermic decomposition (Figure 7a). The thermogram of β‐CD showed a wide endothermic peak at about 90 ~ 100°C (Figure 7b), which could be related to the dehydration of water molecules that bind to β‐CD molecules (Marini et al., 1996). Besides, there was an intensive peak at 313.85°C that derived from melting and endothermic decomposition of β‐CD. All of the peaks at about 83, 127, and 310°C were observed for the case of the physical mixture of IvEO and β‐CD (Figure 7c), which means that DSC curve of the physical mixture of IvEO and β‐CD was a superimposition of individual components of IvEO and β‐CD. Unlike the other three samples, there was no obvious endothermic found at 50 ~ 150°C for β‐CD‐IvEO‐ICs (Figure 7d). As previous report, the inclusion complexes of guest molecules by β‐CD in aqueous solutions resulted in a substantial rearrangement and removal of the water molecules originally solvate to both the β‐CD and guest molecules, and this process also induced the release of water molecules from the β‐CD cavity into the bulk water (Rekharsky M., & Inoue, Y., 1998). Therefore, no endothermic peaks of IvEO and water from β‐CD could be observed at 50 ~ 150°C in β‐CD‐IvEO‐ICs. The endothermic peak at about 165.22°C originally in the IvEO was slightly shifted to a higher temperature of 173.83°C for the inclusion complexes system, which could be explained on the basis of a major interaction which is called intermolecular force between IvEO and β‐CD (Wei et al., 2014). The two endothermic peaks associated with β‐CD were not present in the DSC scan of the β‐CD‐IvEO‐ICs, indicating that a new phase was formed during complexing process.

#### 3.3.4 Thermal stability

**FIGURE 8:**
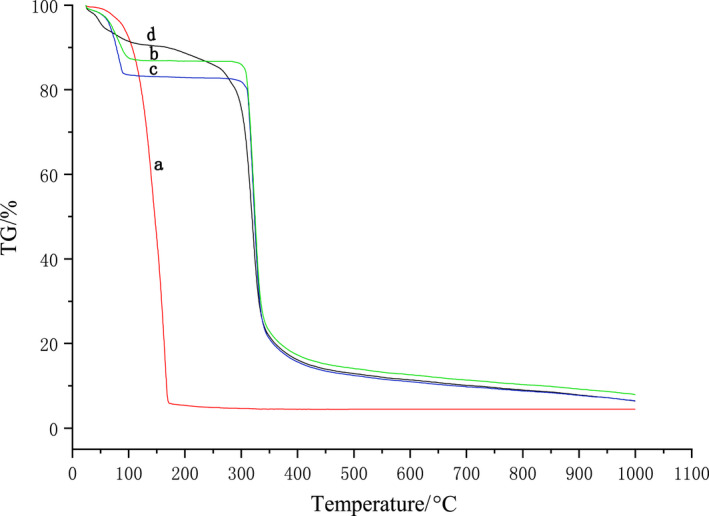
Thermogravimetric (TG) curves of *illicium verum* essential oil (IvEO) (a), β‐cyclodextrin (β‐CD) (b), physical mixture (c), and β‐CD‐IvEO‐ICs (inclusion complexes) (d).

Differences in thermal stability among β‐CD, IvEO, physical mixture, and β‐CD‐IvEO‐ICs were examined by thermogravimetric analysis (TGA) shown in Figure 8. According to the thermogravimetry (TG) curve of IvEO, weight loss after 104.0°C due to volatilization was visibly seen. On the other hand, weight loss could be observed twice at β‐CD. The first time happened at 60 ~ 110°C due to evaporation of high‐energy water molecules which existed in the hydrophobic cavity of β‐CD, and the second occurred at around 302°C due to the thermal decomposition. The TG curve of physical mixture was similar to that of β‐CD since 92% of physical mixture was β‐CD. Compared with physical mixture and β‐CD, a few free oil evaporated. With increase in temperature from 157.0°C, IvEO released slowly from β‐CD cavities. This was attributed to the interaction between IvEO and β‐CD, which improved the thermal stability of inclusion.

### 3.4 Comparison of antioxidative activity between IvEO and β‐CD‐IvEO‐ICs

#### 3.4.1 Ferric reducing antioxidant power

The reducing power (FRAP) of the β‐CD and IvEO, as well as physical mixture and β‐CD‐IvEO‐ICs, was determined by the FRAP assay (Tables 2 and 3). In 10 min, IvEO exhibited the highest reducing power (FRAP) with 144.320 μmol Trolox 100 g^−1^, and the reducing power of anethole was less than IvEO and β‐CD‐IvEO‐ICs. The order in the reducing capacity of samples in 10 min could be observed as well: IvEO > β‐CD‐IvEO‐ICs > anethole > β‐CD. On the other hand, β‐CD‐IvEO‐ICs showed the strongest reducing power with 159.520 Trolox 100 g^−1^ in 1 h. Thus, the order in the reducing capacity of samples in 1 h could be observed as well: β‐CD‐IvEO‐ICs > IvEO > anethole > β‐CD

**TABLE 2:** The reducing power of the β‐cyclodextrin‐*illicium verum* essential oil inclusion complexes (β‐CD‐IvEO‐ICs), IvEO, anethole, β‐CD by using the ferric reducing antioxidant power (FRAP) method in 10 min

Sample	Trolox equivalents (μmol Trolox 100 g^−1^)
β‐CD‐IvEO inclusion complexes	93.120 ± 1.330
IvEO	144.320 ± 1.529
Anethole	41.387 ± 0.251
β‐CD	4.320 ± 0.548

**TABLE 3:** The reducing power of the β‐cyclodextrin‐*illicium verum* essential oil‐inclusion complexes (β‐CD‐IvEO‐ICs), IvEO, anethole, and β‐CD by using the ferric reducing antioxidant power (FRAP) method in 1 h

Sample	Trolox equivalents (μmol Trolox 100 g^−1^)
β‐CD‐IvEO inclusion complexes	159.520 ± 2.308
IvEO	145.920 ± 1.089
Anethole	51.253 ± 0.754
β‐CD	5.920 ± 0.218

Trolox‐equivalent antioxidant capacity of all samples increased with increase in reaction time from 10 min to 1 h. This was attributed to adequate reaction time, which was of benefit for the full reaction between the reductant and Fe^3+^‐TPTZ (2,4,6‐tripyridyl‐s‐triazine). Compared with 10 min, the reducing power of β‐CD‐IvEO‐ICs increased substantially in 1 h reaction time, which could be due to the controlled release of IvEO in inclusion complexes, resulting in stronger antioxidative activities as the reaction time was extended. This phenomenon was in line with those observed in studies (Khatibi et al., 2021; Wang et al., 2021). Interestingly, the reducing power of β‐CD‐IvEO‐ICs was higher than that of IvEO after 1 h reaction time, which could be related to the complexing process.

#### 3.4.2 PTIO radical‐scavenging activity of IvEO and β‐CD‐IvEO‐ICs

As reported, excessive ROS levels are known to be harmful to major biomolecules such as lipid, protein, and nucleic acid in cells and these ROS include singlet oxygen, the hydroxyl radical, the superoxide anion, and hydrogen peroxide (Ghate et al., 2019). Thus, ROS‐scavenging plays an important role in various fields, such as foods, chemical industries, medicine, nutrition, pharmacology, and traditional Chinese medicine (Amorati & Valgimigli, 2015; Foti, 2015; Tai et al., 2011). Compared with nitrogen‐centered radicals, for example, DPPH· (1,1‐diphenyl‐2‐picryl‐hydrazyl radical) and ABTS^+^· (2,2′‐azino‐bis [3‐ethylbenzothiazoline‐6‐sulfonic acid] radical ion), PTIO· (2‐phenyl‐4,4,5,5‐tetramethylimidazoline‐1‐oxyl‐3‐oxide) is a stable, hydrophilic, oxygen‐centered radical, which can evaluate ROS‐scavenging levels (Li, 2017).

**FIGURE 9:**
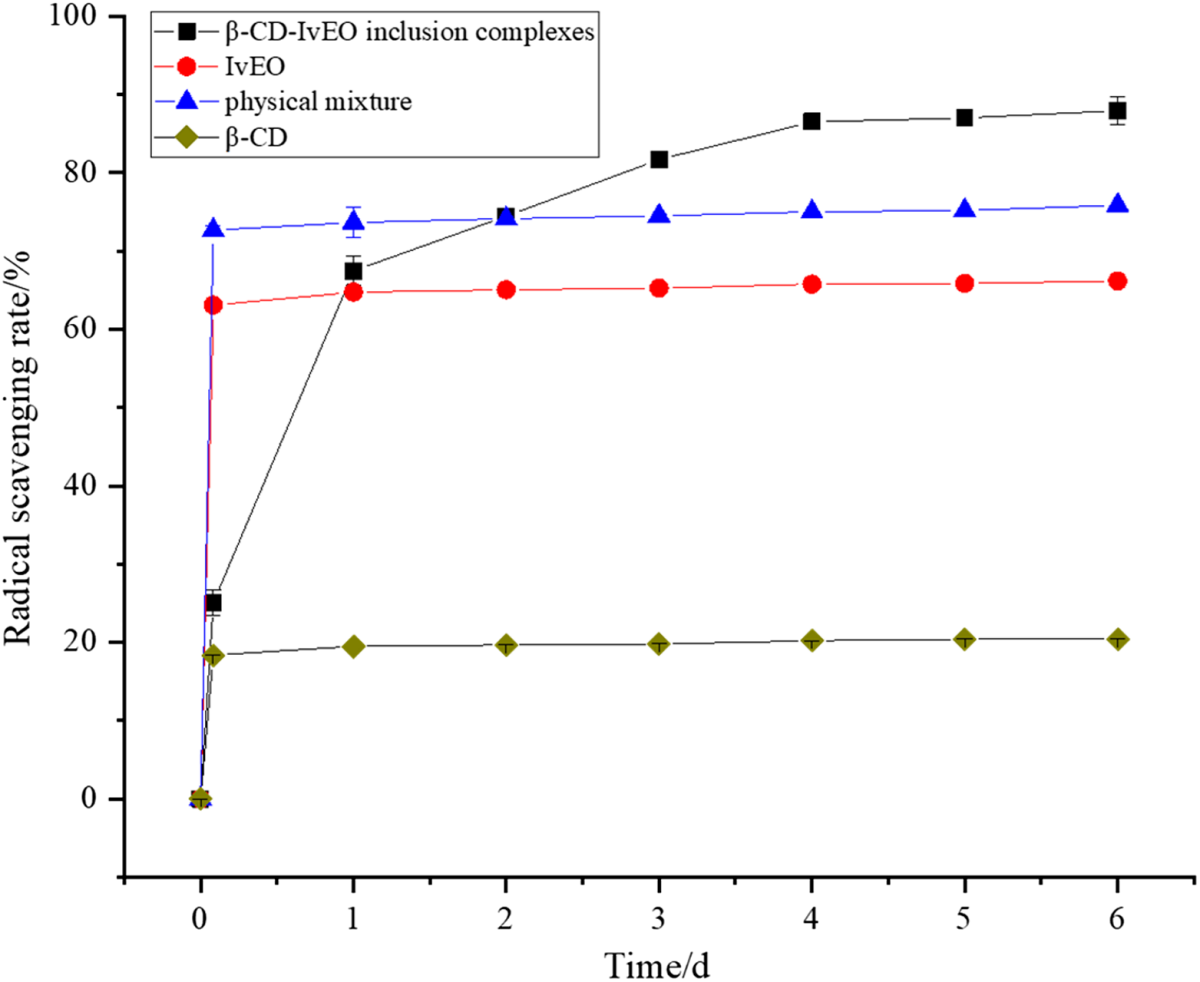
Radical scavenging of β‐cyclodextrin‐*illicium verum* essential oil inclusion complexes (β‐CD‐IvEO‐ICs), IvEO, physical mixture, and β‐CD with phenyl‐4,4,5,5‐tetramethylimidazoline‐1‐oxyl‐3‐oxide (PTIO) method.

As shown in Figure 9, β‐CD‐IvEO‐ICs, IvEO, physical mixture, and β‐CD had certain radical‐scavenging capacity. The radical‐scavenging rates of IvEO, physical mixture, and β‐CD were basically saturated within 2 h. But the PTIO‐ scavenging rate of β‐CD‐IvEO‐ICs reached 25.06%, 67.47%, 74.46%, 81.69%, 86.51%, 86.99%, and 87.95% with 2 h, 1 day, 2 days, 3 days, 4 days, 5 days, and 6 days reaction time, respectively, which presented the tendency of rapid increase first and then slow increase. This suggested that the β‐CD‐IvEO‐ICs could scavenge PTIO in a slow and gradual process due to which the liquid core materials can constantly release to external environment through solution and diffusion. The ability of all samples scavenging PTIO followed the order: β‐CD‐IvEO‐ICs > physical mixture > IvEO> β‐CD. This finding meant that the stability of guest molecule was improved during the complexing process so that it can react with PTIO· completely.

In the radical‐scavenging rate curves (Figure 9), the physical mixture showed higher PTIO· scavenging capacity than IvEO and β‐CD also had the ability of scavenging radical. But β‐CD had weak reducing power. This phenomenon could be attributed to the difference between PTIO· and Fe3 + ‐TPTZ (2,4,6‐tripyridyl‐s‐triazine) as core materials. A study showed that β‐CD can selectively bind aromatic compounds with suitable shape and size to form supramolecular system (Jiang, 2011). PTIO, as an aromatic derivation, was probably partially complexed in β‐CD during scavenging process, which causes a drop in the concentration of PTIO· solution. It is difficult for the radical that was complexed to react with the outside world so that it could be considered a way to scavenge radical, which meant that β‐CD had radical‐scavenging capacity in appearance.

### 3.5 Comparison of antibacterial activity between IvEO and β‐CD‐IvEO‐ICs

#### 3.5.1 DGZ of IvEO and β‐CD‐IvEO‐ICs (diameter of growth zone)

The IvEO was widely used as food additives and traditional medicines because of its potent antimicrobial, anti‐inflammatory, and antioxidant properties. Therefore, it was neccessary for IvEO and β‐CD‐IvEO‐ICs to evaluate their antibacterial activities of food‐borne bacteria. The DGZ of IvEO and β‐CD‐IvEO‐ICs could be seen in Table 4, which suggested that IvEOs exhibit antibacterial activity against all the tested food‐borne bacteria, during the 10‐day storage period.

**TABLE 4:** Growth zone diameter of *illicium verum* essential oil (IvEO) and β‐cyclodextrin‐IvEO inclusion complexes (β‐CD‐IvEO‐ICs) against tested microorganisms (mm)

Sample	Growth zone diameter (mm)，disk diameter 6.0 mm was included									
	Storage time (day)									
	1	2	3	4	5	6	7	8	9	10
*Escherichia coli*										
IvEO	6.6 ± 0.1	16.6 ± 0.1	23.4 ± 0.2	27.4 ± 0.6	28.7 ± 0.4	34.8 ± 0.4	38.0 ± 0.7	41.6 ± 1.2	52.3 ± 1.1	59.0 ± 0.4
β‐CD‐IvEO inclusion complexes	6.0 ± 0.0	8.0 ± 0.2	13.3 ± 0.2	14.6 ± 0.8	16.9 ± 0.2	20.2 ± 0.4	23.5 ± 0.3	25.6 ± 0.4	32.7 ± 0.3	36.4 ± 0.2
Sterile water	14.6 ± 0.4	23.6 ± 1.2	30.6 ± 1.0	36.8 ± 0.5	41.0 ± 0.3	45.0 ± 1.0	50.5 ± 0.4	54.0 ± 0.8	61.4 ± 0.4	64.6 ± 1.4
*Bacillus subtilis*										
IvEO	6.6 ± 0.0	13.6 ± 0.5	22.0 ± 0.2	30.0 ± 0.8	36.7 ± 0.6	39.2 ± 1.2	43.7 ± 0.6	46.6 ± 1.1	49.1 ± 0.4	52.0 ± 0.8
β‐CD‐IvEO inclusion complexes	6.0 ± 0.0	6.0 ± 0.0	9.6 ± 0.5	13.8 ± 0.4	16.7 ± 0.2	20.2 ± 0.6	24.9 ± 0.3	26.4 ± 0.8	28.6 ± 0.3	31.8 ± 0.6
Sterile water	7.4 ± 0.1	18.8 ± 1.5	33.8 ± 0.8	41.0 ± 1.6	49.1 ± 0.4	58.6 ± 0.6	61.9 ± 0.8	67.4 ± 1.0	73.9 ± 1.0	83.6 ± 0.8
*Staphylococcus epidermidis*										
IvEO	6.7 ± 0.1	17.6 ± 0.2	34.0 ± 0.9	42.7 ± 0.8	56.0 ± 1.3	60.6 ± 2.0	79.5 ± 1.4	81.0 ± 1.2	83.3 ± 0.9	85.0 ± 1.7
β‐CD‐IvEO inclusion complexes	6.3 ± 0.1	7.4 ± 0.6	12.8 ± 0.2	20.8 ± 0.4	30.3 ± 0.2	36.6 ± 0.2	39.9 ± 0.8	54.6 ± 1.4	62.3 ± 1.4	66.2 ± 1.3
Sterile water	14.0 ± 0.6	18.6 ± 0.8	36.6 ± 1.3	48.6 ± 1.6	72.6 ± 2.4	88.6 ± 1.8	90.0 ± 0.0	90.0 ± 0.0	90.0 ± 0.0	90.0 ± 0.0
*Staphylococcus aureus*										
IvEO	6.3 ± 0.1	9.6 ± 0.3	11.7 ± 0.8	13.1 ± 0.4	18.2 ± 1.0	21.6 ± 1.0	32.3 ± 0.6	41.2 ± 0.8	54.7 ± 1.3	60.2 ± 1.1
β‐CD‐IvEO inclusion complexes	7.4 ± 0.2	8.4 ± 0.4	10.2 ± 0.4	10.8 ± 0.4	12.8 ± 0.4	13.0 ± 0.5	13.2 ± 0.2	15.4 ± 0.4	21.7 ± 1.3	32.6 ± 0.6
Sterile water	17.8 ± 0.4	33.0 ± 1.2	48.5 ± 2.8	52.4 ± 1.4	65.7 ± 2.6	71.3 ± 2.5	74.0 ± 1.1	75.6 ± 1.0	78.5 ± 1.6	81.2 ± 0.9

*Note*: Values are the mean ± SD.

Each value is the mean of three replicate experiments.

At the beginning of the culture period, both IvEO and β‐CD‐IvEO‐ICs demonstrated strong antimicrobial activity against all the tested bacteria, with the DGZ of less than 10 mm for *Staphylococcus aureus*, 14 mm for *Bacillus subtilis*, 17 mm for *Escherichia coli*, and 18 mm for *Staphylococcus epidermidis*. The strongest antibacterial effect of IvEOs was observed against *E. coli*, followed by *S. aureus*, *B. subtilis*, and *S. epidermidis* in 2‐day culture time.

With increased culture time, the antibacterial activities of IvEO and β‐CD‐IvEO‐ICs were found to weaken significantly. However, β‐CD‐IvEO‐ICs demonstrated a stronger and more long‐lasting antibacterial property than IvEO. Futhermore, β‐CD‐IvEO‐ICs demonstrated lower DGZ values than IvEO against all the tested food‐borne bacteria. This phenomenon could be attributed to the loss of volatile components of IvEOs over time, which suggested that strong antibacterial activity of IvEO was conserved for a long time due to the less loss embedded IvEO whose volatile components were protected (Marino et al., 2001).

In addition, bacteria might build up a tolerance to essential oil, which means that when IvEO possessed an inhibitory effect to bacteria but not enough to kill them, they might develop a tolerance to IvEO and transmit the information to the offspring. For *S. epidermidis* and *E. coli*, the 10‐day culture DGZ of IvEO was shown to be comparable to the DGZ of sterile water. The phenomenon, which presented inhibition first then growth normally, was probably because bacteria had tolerance to IvEO for generations. However, the 10‐day culture DGZ of β‐CD‐IvEO‐ICs was smaller than sterile water for four targeted bacteria. This was probably because bacteria would not develop a strong tolerance when they were exposed to IvEO with low concentration due to the controlled release of complexed IvEO. Besides, β‐CD‐IvEO‐ICs could inhibit the growth of bacteria for generations as the concentration increased which means inhibition of β‐CD‐IvEO‐ICs on bacteria is a long‐term and effective process.

#### 3.5.2 Excessive ROS production in IvEO‐treated and β‐CD‐IvEO‐ICs‐treated bacteria

The methodology used to cultivate bacteria was described in section 2.7.3, which presents that the higher addition amount of IvEOs, the lower was the bacterial count. Futhermore, when IvEOs content added was 48 μl, plates of β‐CD‐IvEO‐ICs demonstrated tiny amounts of bacteria and only the growth of *S. aureus* could be seen in the plates of IvEO.

**FIGURE 10:**
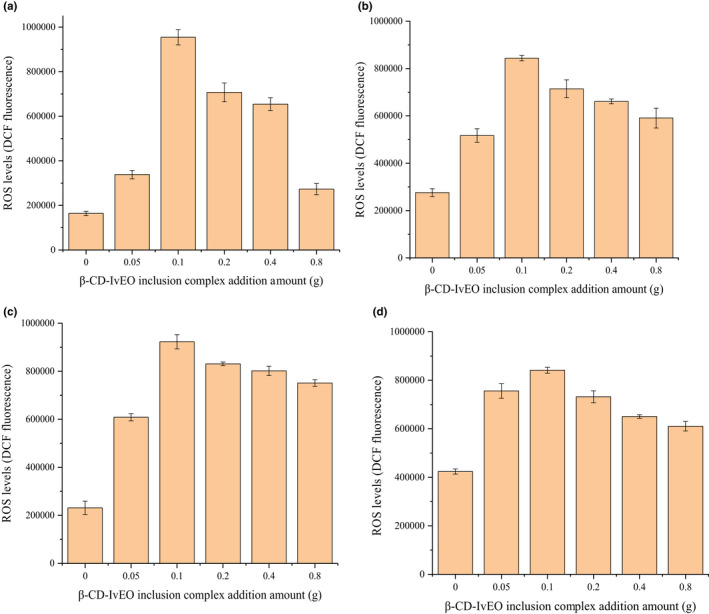
Production of reactive oxygen species (ROS) of *Escherichia coli* (a), *Bacillus subtilis* (b), *Staphylococcus epidermidis* (c), and *Staphylococcus aureus* (d) under different treatment concentrations with β‐cyclodextrin‐*illicium verum* essential oil inclusion complexes (β‐CD‐IvEO‐ICs) in 24 h.

As illustrated in Figure 10 and Table 5, bacterial dichlorofluorescein (DCF) fluorescence displayed a trend of increasing to a maximum and then decreasing under different treatment concentrations with IvEO and β‐CD‐IvEO‐ICs, reaching the highest value at addition of 0.1 g. It is noted that DCF fluorescence is proportional with ROS levels. Meanwhile, weak fluorescence signals were detected in the negative control group probably because bacteria produced endogenous ROS during its metabolic process, which are in line with Tan et al. (Lijun et al., 2021). The ROS levels increased with increased addition of IvEOs, which could be explained by the fact that small molecules from IvEOs, deposited in bacterial cell wall in quantities with increase in the addition of IvEOs, can program bacteria to produce excessive ROS. The excessive oxidative ROS may damage the bacteria and cause bacterial lysis to death (Lijun et al., 2021). As mentioned above, the mechanism of IvEOs was similar to antibiotics which stimulate the production of highly deleterious hydroxyl radicals in bacteria through drug–target interaction (Kohanski et al., 2007).

**TABLE 5:** Production of reactive oxygen species (ROS) of *Escherichia coli* (a), *Bacillus subtilis* (b), *Staphylococcus epidermidis* (c), and *Staphylococcus aureus* (d) under different treatment concentrations with IvEO and β‐CD‐IvEO inclusion complexes in 24 h

Sample	IvEOs equivalents					
	0 μl	3 μl	6 μl	12 μl	24 μl	48 μl
*Escherichia coli*						
IvEO	16.352 ± 0.970	31.183 ± 2.620	91.077 ± 3.193	30.557 ± 0.898	39.093 ± 1.822	‐
β‐CD‐IvEO inclusion complexes	16.352 ± 0.970	33.810 ± 1.882	95.438 ± 3.425	70.711 ± 4.202	65.453 ± 2.873	27.329 ± 2.516
*Bacillus subtilis*						
IvEO	27.614 ± 1.641	37.713 ± 3.510	41.498 ± 1.036	50.945 ± 1.465	44.156 ± 2.709	‐
β‐CD‐IvEO inclusion complexes	27.614 ± 1.641	51.733 ± 2.854	84.389 ± 1.100	71.451 ± 3.777	66.134 ± 1.043	59.093 ± 4.192
*Staphylococcus epidermidis*						
IvEO	23.101 ± 2.750	53.709 ± 1.264	70.660 ± 3.727	89.794 ± 2.660	77.005 ± 5.320	‐
β‐CD‐IvEO inclusion complexes	23.101 ± 2.750	60.859 ± 1.477	92.298 ± 2.939	83.090 ± 0.778	80.178 ± 1.965	75.127 ± 1.342
*Staphylococcus aureus*						
IvEO	42.382 ± 1.062	54.468 ± 2.386	59.066 ± 0.956	69.621 ± 3.122	78.744 ± 3.401	72.515 ± 2.286
β‐CD‐IvEO inclusion complexes	42.382 ± 1.062	75.605 ± 2.982	84.095 ± 1.195	73.180 ± 2.436	65.038 ± 0.741	61.067 ± 1.939

*Note*: Values are the mean ± SD.

Each value is the mean of three replicate experiments.

Surprisingly, the ROS levels were relatively lower when IvEOs increased to a certain extent. This could be attributed to excessive oxidative ROS, which broke the cell structure and increase the bacterial membrane permeability. It can be predicted from section 3.4.2 that IvEO and its inclusion complexes had ROS‐scavenging capacity. Moreover, a study reported that the diffusion ability of an EO through the wall (cell membrane) of a bacterium is important in terms of its antibacterial effect (Mutlu‐Ingok et al., 2020). In summary, this phenomenon could be explained by the fact that small molecule active ingredients of essential oil can enter into bacteria and scavenge ROS when the membrane permeability of bacteria increases to a certain extent. This led to a result that DCFH, produced by hydrolysis of esterase, cannot be oxidized into DCF, which causes a drop in DCF fluorescence.

The differences of bacterial ROS production level between IvEO and β‐CD‐IvEO inclusion complexes were measured by 2′,7′‐dichlorofluorescein diacetate (H_2_DCF‐DA). As shown in Table 5, the DCF fluorescence of β‐CD‐IvEO inclusion complexes was significantly higher than that of IvEO, which is probably due to its increased water solubility that led to increased contact area between the bacteria and essential oil, thus improving antimicrobial activity and stimulating the bacteria to produce more excessive ROS (Wang et al., 2019). A study also showed that lethal action derives from stimulation of a self‐amplifying accumulation of ROS that overwhelms the repair of primary damage (Hong et al., 2019). The result is in line with section 3.5.1, which means that complexed IvEO can induce more excessive ROS production in bacteria than IvEO equivalents due to the higher water solubility and stability. In addition, for *E. coli*, *S. aureus*, *B. subtilis*, and *S. epidermidis*, the minimum DCF fluorescence was 33.55%, 86.67%, 85.76%, and 92.09% of maximum DCF fluorescence, respectively, in the IvEO‐treated group and 28.64%, 70.02%, 81.40%, and 72.62% in the IvEO‐ICs‐treated group. It may be attributed to stronger ROS‐scavenging capacity for IvEO‐ICs that were taken into the bacteria, which is in line with section 3.4.2.

## 4 CONCLUSION

Vaporized IvEO was encapsulated through gas–liquid embedding method with β‐CD as wall materials and the IvEO‐β‐CD‐ICs exhibited the high EE of 84.55 ± 2.31%. Results of FTIR, DSC, and TG measurements demonstrated that the vaporized IvEO had encapsulated into IvEO‐β‐CD‐ICs and that the stability of IvEO‐β‐CD‐ICs was improved. In addition, the antioxidative activities of IvEO‐β‐CD‐ICs were slightly decreased compared with that of IvEO during the early period of reaction, whereas IvEO‐β‐CD‐ICs demonstrated long‐lasting antioxidative activities and antibacterial activities against *E. coli*, *B. subtilis*, *S. epidermidis*, and *S. aureus* during the whole period. Therefore, we suggest that IvEO‐β‐CD‐ICs could be used as a natural preservative. However, further studies are required to improve the quality of food with IvEO‐β‐CD‐ICs during storage and investigate the preservation effects on tested food.

## FUNDING INFORMATION

This work was supported by the Forestry Scientific and Technology Innovation Project of Guangdong Province (2020KJCX010), Science & Technology Planning Project of Guangzhou City (202103000078), and Science & Technology Planning Project of Guangdong Province (19ZK0364).

## CONFLICT OF INTEREST

There are no conflicts of interest.

## ETHICAL APPROVAL

This study does not involve any human or animal testing.

## Data Availability

The data sets generated during and/or analyzed during the current study are available from the corresponding author on reasonable request.
